# The cartonectin levels at different stages of chronic kidney disease and related factors

**DOI:** 10.1080/0886022X.2018.1561373

**Published:** 2019-02-07

**Authors:** Yasemin Coskun Yavuz, Konca Altınkaynak, Can Sevinc, Saime Ozbek Sebin, Idris Baydar

**Affiliations:** a Department of Nephrology, Faculty of Medicine, Selcuk University, Konya, Turkey;; b Erzurum Regional Research and Training Hospital, Erzurum, Turkey;; c Department of Physiology, Faculty of Medicine, Ataturk University, Erzurum, Turkey

**Keywords:** Adiponectin, cartonectin, CKD, CTRP, hemodialysis

## Abstract

**Introduction:** Cartonectin was defined as a new adipokine released from rat and human adipocyte tissues, which is also known as CORS 26 or CTRP3 protein. Although there are several studies investigating the effects of cartonectin with obesity, anti-inflammatory mechanisms, and cardioprotective effects, there is no study about the effects of cartonectin in patients with chronic kidney disease yet. We aimed to investigate cartonectin levels in predialysis and dialysis patient groups, in other words, at different stages of chronic kidney disease, by comparing with the control group. In addition, we aimed to discuss the probable causes of the differences between the patient groups that would be determined, together with the factors that might be effective.

**Methods:** A total of 150 patients, including 47 hemodialysis patients, 73 predialysis CKD patients, and 30 healthy individuals were enrolled in the study. Serum cartonectin levels were determined by using enzyme-linked immunosorbent assay (ELISA) method.

**Findings:** Serum cartonectin levels were found to be significantly higher in the hemodialysis patient group compared to predialysis group and healthy individuals (*p* < 0.01). Furthermore, serum cartonectin levels were found to be negatively correlated with GFR, BMI, glucose, LDL, and platelet levels, whereas a positive correlation was observed with creatinine levels.

**Discussion:** In our study, we found that the cartonectin levels increased as GFR decreased and were significantly higher in hemodialysis patients. Cartonectin is structurally closely related to adiponectin. It is remarkable that the level of cartonectin is also high in hemodialysis patients, like adiponectin.

## Introduction

Adipokines are released from the adipose tissue. In recent years, CTRP (C1q/TNF-related protein) family of 15 members has been added to this adipokine family. Cartonectin, which is also known as cartducin, CTRP3 and CORS-26, is a member of this family; it has anti-inflammatory and cardioprotective effects similar to adiponectin. Cartonectin levels were studied in patients with obesity, insulin resistance, type-2 diabetes mellitus; however, conflicting results have been found [1,3]. Cartonectin levels were found to be reduced in patients with angina pectoris and acute coronary syndrome [4,5].

We aimed to investigate cartonectin levels in patients at predialysis and hemodialysis stages of chronic kidney disease in whom cartonectin levels had not been studied before and to observe various parameters that can affect cartonectin levels in these patients.

## Materials and method

The ethical approval was obtained from the Ethical Committee of Erzurum Regional Training and Research Hospital. Verbal and written consents were obtained from all patients before the study.

## Subjects

A total of 150 individuals consisting of 47 hemodialysis patients, 73 predialysis patients, and 30 healthy controls were included in the study. Exclusion criteria were the presence of type-2 diabetes mellitus, coronary artery disease (in the past, myocardial infarction, angina pectoris, coronary interventional procedures were excluded from the study), infection, chronic liver disease or malignancy. Patients under 18 years old were also excluded from the study.

The blood samples were obtained after an 8-h fasting period, and biochemical parameters were studied along with the complete blood count and C-reactive protein (CRP) levels. Blood samples were obtained just before initiation of the hemodialysis in the hemodialysis patient group. Blood samples for studying cartonectin levels were centrifuged immediately after obtaining the samples. The separated serum samples were stored at −80 °C. BMI was calculated by using the body weight (kg)/height^2^ (meter^2^) formula after measuring the weight and height. The CKD-EPI formula is used for GFR calculation.

## Analysis of cartonectin

Blood samples were centrifuged at +4 C and 4000 rpm for 10 min. The serum samples were transferred to Eppendorf tubes after aliquoting. The samples were stored at −80 °C until the day that the study was performed.

The serum cartonectin levels were measured by using Human Cartonectin Elisa Kit (Bioassay Technology Laboratory, Cat # E3300Hu, China) according to the directions provided by the manufacturer. The analysis was performed using BioTek PowerWaveST microplate spectrophotometer (USA) device, and the cartonectin levels were recorded as ng/ml. Standard curve ranges were 0.2 to 60 ng/ml, sensitivity was 0.09 ng/ml. The intra-assay coefficients of CTRP3 were <%8, and the inter-assay coefficients were <%10.

### Statistical analysis

Data were analyzed by using SPSS 17.0 program. *p* < 0.05 was defined as statistically significant. Mean ± standard deviation was used for numerical variables. Student t-test, Mann–Whitney U-test, Kruskal–Wallis test and Chi-square test were used for statistical analysis. Spearman bivariate correlation analysis test was used for correlation analysis.

## Results

A total of 150 participants, consisting of 47 hemodialysis patients (group 1), 73 predialysis patients (group 2), and 30 healthy individuals (group 3) were included in the study. Eighty-six individuals were male (57.3%), and the remaining 64 individuals (42.7%) were female. The youngest and the oldest participants were 21 and 75 years old, respectively. There was no significant difference between three groups in terms of age, weight, BMI, together with WBC, platelet count, and hemoglobin, glucose, triglyceride levels (Table 1).

**Table 1.** Comparison of groups.

	Group 1 (hemodialysis) *N* = 47	Group 2 (predialysis CKD) *N* = 73	Group 3 (control) *N* = 30	*p*-Value
Age (year)	51 ± 11.5	51 ± 11.1	46.5 ± 8.8	0.4
Cartonectin (ng/ml)	37.2 ± 17.8	24.6 ± 14.9	27.2 ± 14.2	0.01*
GFR (ml/min)	4.7 ± 1.7	30 ± 15	101 ± 10.2	<0.001*
BUN (mg/dL)	81.2 ± 20	42 ± 19.2	13.6 ± 3.3	<0.001*
Creatinine (mg/dL)	10.5 ± 2	2.8 ± 1.6	0.77 ± 0.1	<0.001*
Glucose (mg/dL)	87 ± 9.5	88 ± 8	86.3 ± 10.2	0.17
LDL (mg/dL)	80 ± 29	118 ± 37.6	122 ± 43.8	0.004*
TG (mg/dL)	145 ± 67	158 ± 80	133 ± 68.6	0.62
HGB (g/dL)	10.7 ± 1.6	13.8 ± 2.3	15.3 ± 1.5	<0.001*
PLT (x10^3 u/L)	182 ± 68.5	290 ± 35	286 ± 70	0.07
WBC (x10^3 u/L)	7.4 ± 2.1	7.9 ± 2.2	7.2 ± 2	0.86
Weight (kg)	66 ± 12.8	69.6 ± 9.4	72.7 ± 13	0.03*
BMI (kg/m^2^)	25.3 ± 5.8	25.5 ± 3.3	25.8 ± 3.3	0.87

*p < 0.05.

When the groups were compared regarding cartonectin level, it was found to be statistically significantly higher in the hemodialysis patient group (37.2 ± 17.8) than the predialysis group (24.6 ± 14.9) and the control group (27.2 ± 14.2) (*p* < 0.01) (Table 1 and Figure 2).

**Figure 1.**
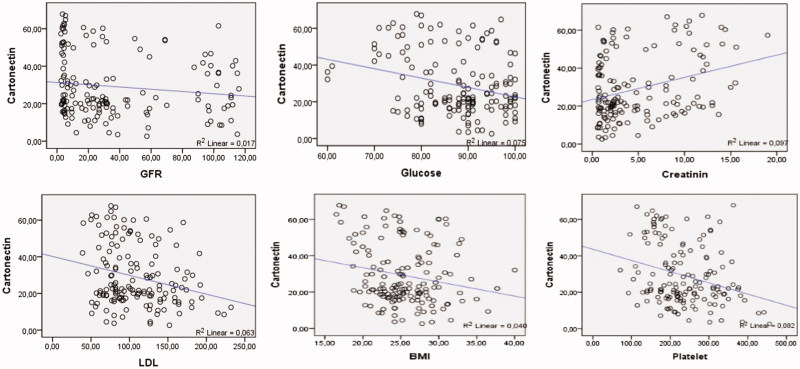
Correlation data.

The correlation of cartonectin level with demographic and biochemical parameters was also analyzed. A negative correlation was observed between GFR and cartonectin level (*p* = 0.009, *r* = −0.21). Negative correlation was observed between cartonectin and glucose (*p* = 0.001, *r* = −0.27). There was a positive correlation between cartonectin and creatinine (*p* < 0.001, *r* = 0.31). There was a negative correlation between cartonectin and LDL (*p* = 0.002, *r* = −0.25). There was a negative correlation between cartonectin and platelet count (*p* = 0.001, *r* = −0.26). There was a negative correlation between cartonectin and BMI (*p* = 0.01, *r* = −0.20) (Figure 1). There was no significant correlation between cartonectin level and gender, age, weight and BUN parameters.

**Figure 2.**
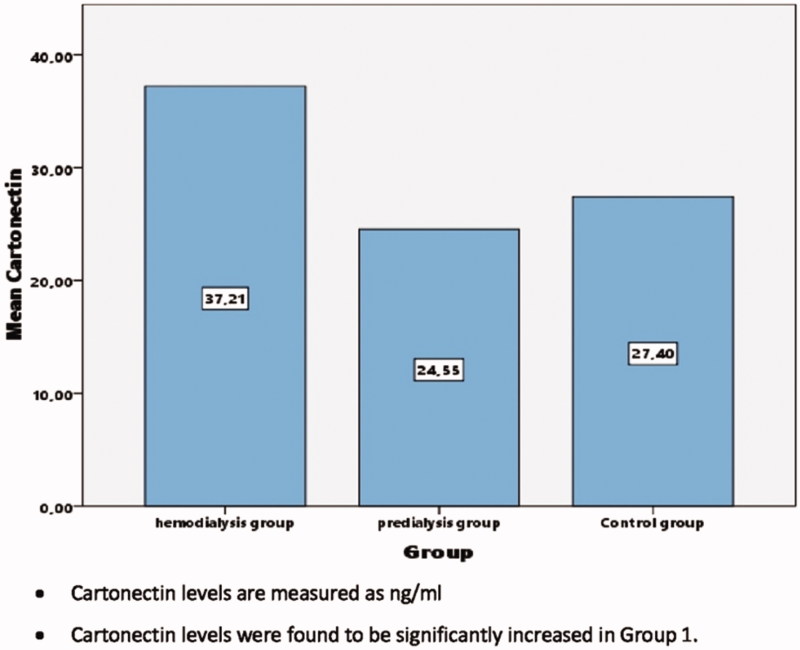
Cartonectin levels of groups.

## Discussion

Cartonectin is a member of a recently identified adipokine family named CTRP, which consists of 15 members. It was first cloned in 2001 and studied in plasma in 2007. It is expressed in lungs, kidneys, spleen, testis, macrophages, heart, intestines, liver, and muscle tissue, as well as adipose tissue [17]. The effects of cartonectin on growth and development, inflammation, hepatic lipid metabolism and cardiovascular system have been defined. CTRP3 was also detected during the development of chondrocytes and cartilage; the names of cartonectin and cartducin depend on this detection. It stimulates osteogenic and chondrogenic proliferation and also inhibits osteoclasts. It is the closest functional analog of adiponectin in the CTRP family [1]. Cartonectin has an anti-inflammatory effect. It inhibits TLR (Toll-like receptor) and Nkf β signal pathways along with IL-6 and TNF-alfa. Furthermore, it stimulates the expression of adiponectin in human adipocytes. While ablation of cartonectin leads to an increase in proinflammatory cytokines, it also decreases the expression of adiponectin [2]. Exogenous administration cartonectin helps to improve post-ischemic cardiac functions and reduces the apoptosis rate [3]. Based on these findings, it may be considered that CTRP3 can be used in the treatment of inflammatory diseases.

Cartonectin was found to be significantly decreased in rats with diabetic cardiomyopathy, and its exogenous administration was determined to be therapeutically effective [3]. The effect of cartonectin in patients with diabetes mellitus is a conflicting subject. Choi et al. reported that cartonectin levels were increased in patients with diabetes mellitus [6], whereas Ban et al. reported reduced levels [7].

A similar conflict is also present in obese patients. Wolf et al. reported significantly decreased cartonectin levels in obese patients [8], whereas Wagner et al. reported significantly increased cartonectin levels in male patients with obesity [9]. Deng et al. reported that cartonectin levels were decreased in patients with obesity and hypertension [10]. Flehmig et al. reported that cartonectin levels were significantly increased after metformin treatment [11]. Li et al. found that cartonectin levels were significantly decreased in diabetic rats [12]. Ban et al. reported that cartonectin levels were increased in type-2 DM patients. They also reported a negative correlation of serum cartonectin level with glucose and CRP levels [7]. Similarly, we also found a negative correlation between glucose and cartonectin levels.

Tan et al. reported that adipose tissue cartonectin levels were lower in patients with PCOS. Additionally, they found negative correlations between BMI, glucose, insulin, LDL, TG, and CRP. They determined that serum cartonectin level increased in this patient group following 6-month metformin treatment [13]. Similarly, we also found that cartonectin was negatively correlated significantly with BMI, glucose, and LDL levels. In their study conducted in 2001, Choi et al. reported a negative correlation between creatinine clearance and cartonectin level and a positive correlation between creatinine and cartonectin levels. We also obtained similar results in our study. However, the patient group in the study of Choi et al. did not include patients with chronic kidney disease [6].

Schaffler et al. reported that cartonectin administration resulted in adiponectin secretion [14]. In their study consisting of 368 patients, Liu et al. reported that adiponectin, resistin, MCP-1 and adipsin levels were significantly increased in dialysis patients. They asserted that the decreased renal clearance was related to increased adipokine levels [15]. We have found no study conducted prior to our study in which the cartonectin level was investigated in patients with chronic kidney disease. Adiponectin and cartonectin are 36% homologous to each other in alignment in humans [16]. As found in the studies conducted with adiponectin, we found that the level of cartonectin, which has the closest structural similarity to adiponectin, was increased in the hemodialysis patient group when compared to the control and predialysis groups, which were similar regarding BMI and weight in our study, even in the presence of insulin resistance and high prevalence of cardiovascular disease. We also were unable to explain exactly why cartonectin is elevated with the reduction of creatine clearance. We can assert that adiponectin and cartonectin have parallel effects on the hemodialysis patient group and also that a negative correlation is present between creatinine clearance and cartonectin, similar to adiponectin. In this group of patients, high levels of adiponectin have been tried to be explained by several mechanisms. Adiponectin is excreted via the kidneys. It may be suggested that there is a negative correlation between GFR and adiponectin and that it is high in patients with hemodialysis, which is not proven. Another explanation is the increased adiponectin level as a compensatory response to the increase in inflammation in ESRD. Another explanation is the adiponectin resistance due to the uremic proinflammatory circulation [18,19]. Okuno and colleagues found that increased adiponectin levels in hemodialysis patients were associated with decreased bone mineral density [20]. Based on the structural similarity between adiponectin and cartonectin, cartonectin elevation in the hemodialysis group may be related to this reason. However, further work is needed on this topic.

## Conclusion

It is difficult to claim that cartonectin and adiponectin levels increase in hemodialysis patient groups when the structural and functional similarities of these two molecules are taken into consideration, thus necessitating support with further studies having larger samples.
